# Quality of Healthcare in Acute Pediatric Care Unit in a Tertiary Hospital in Tanzania: A Case of Muhimbili National Hospital

**DOI:** 10.3389/fped.2020.00496

**Published:** 2020-08-27

**Authors:** Aleya Z. Remtullah, Nathanael Sirili, Amani Anaeli, Augustine Massawe, Karim Manji, Bruno F. Sunguya

**Affiliations:** ^1^Department of Paediatrics, Muhimbili University of Health and Allied Sciences, Dar-es-Salaam, Tanzania; ^2^Department of Development Studies, School of Public Health, Muhimbili University of Health and Allied Sciences, Dar-es-Salaam, Tanzania; ^3^Department of Community Health, Muhimbili University of Health and Allied Sciences, Dar-es-Salaam, Tanzania

**Keywords:** quality of care, structure, process, outcomes, level of satisfaction

## Abstract

**Background:** Quality of care in a critical care unit is vital for the outcomes of critically ill people and especially children, who are more at risk. Although evidence is mixed, only a handful remains documented about the role of quality of care among children in the context of tertiary hospitals of low-income countries such as Tanzania. This study therefore assessed the quality of healthcare in Acute Pediatric Care Unit (APCU) at Muhimbili National Hospital in Tanzania over 3 months.

**Methodology:** This mixed method cross sectional study employed both qualitative and quantitative approaches to gather data from 107 participants that included caregivers of children admitted, and healthcare providers in APCU at MNH. Components of the Donabedian model were used to assess quality of care. Descriptive analyses was conducted for quantitative data while thematic analyses was conducted for qualitative data.

**Results:** A total of 24 (26.7%) of the children admitted in APCU died in the 3-month period of data collection. Of them, 41.7% died during the first 24 h of admission. The median duration of APCU admission was 5 days. Despite the noted challenges, most of the caregivers were very satisfied 34 (37.8%) or satisfied 22 (24.4%) with the quality of services provided. The physical setting in APCU had the basic requirements for management of critically ill children but was insufficient in infrastructure; healthcare providers trained in critical care and updated treatment guidelines amongst others. We noted inadequacy in on-job training of health workers, feedback process, and obvious delays in the referral system.

**Conclusions and Recommendations:** Although one in four children admitted in the APCU at MNH died, the overall quality of care in this tertiary referral hospital was modest as it achieved the minimum acceptable standards. To enhance quality of care, it is vital to improve infrastructure, update treatment guidelines, train staff in critical care and improve the feedback process especially during emergencies and deaths.

## Introduction

Efforts to address child mortality has met significant success in Tanzania like in other developing countries (1). The country had early wins in meeting the Millennium Development Goal number four, aimed at reducing child mortality. Such gains however, vary across the regions and contexts within the country. Despite the overall success, children with severe illnesses continue to succumb from the adverse outcomes owing to poor quality of care even when they reach tertiary health facilities. Poor health systems including poor infrastructure, human resources skills, and lack of essential medicine and supplies, play a significant role in such a challenge.

Magnitudes of mortality within intensive care units varies with countries and regions ranging from 5.4% in Europe to 40% in African countries (2). Unpublished report from Muhimbili National Hospital, the highest level tertiary hospital in Tanzania, revealed the mortality rates in the Acute Pediatric Care Unit (APCU) as follows: in 2014 (41.4%), in 2015 (39.2%), and in 2016 (45.3%). Majority of these deaths were within the first 24 h of admission. Quality of care is an essential component to ensure children with severe illness survive within the first 24 h. However, evidence remains scanty in the country, as well as areas with similar context as in Tanzania.

The Ministry of Health Community Development Gender Elderly and Children (MOHCDGEC) in Tanzania, rated the overall quality of care as poor with several concerns raised in 2010 (3). These included the availability of standard treatment guidelines, essential medicines, equipment, availability of appropriate and adequate human resources, and standards for qualified staff amongst others (3). Several studies have examined quality of care provided to children, however, most of them focused on parents and patient satisfaction (4–6), nurses' perspectives in critical care and trauma (7), and inpatient care (4–7), but very little is known about quality of care in children admitted in critical care unit in tertiary facilities. This study therefore aimed to assess the quality of healthcare in APCU at Muhimbili National Hospital (MNH) in Tanzania.

## Materials and Methods

### Design and Setting

This cross sectional study adopted mixed methods using both qualitative and quantitative approaches (8). Quality of care was assessed using the Donabedian model. Structural component was assessed qualitatively using observation and quantitatively using the semi-structured questionnaires while the process component was evaluated qualitatively using observation and Key Informant Interviews (KIIs), and quantitatively using semi-structured questionnaires. The outcome component of Quality of Care (QOC) was examined quantitatively using document review.

This study was conducted at MNH, a national tertiary, and teaching hospital in Dar-es-Salaam, Tanzania. MNH provides outpatient, emergency, and in-patient services to more than 1,000 patients per week (9). This hospital was chosen owing to the presence of an APCU in the pediatric department providing acute care and monitoring of very sick children. In addition, MNH receives patients from different parts of Tanzania, thus allowing a diversity of information to be collected (9).

The APCU was established in 2003/2004 because, most children that were brought to MNH, passed through a casualty unit where they were seen by general practitioners before being dispatched to the wards. There were minimal interventions done for a very sick child causing 70–90% of deaths within 24–48 h of admission. The most common cause of death being respiratory distress.

The launched APCU contained all the emergency medications (e.g., dextrose 10%, i.v. diazepam), infusion pumps, oxygen central lines and concentrators, trained staff, and permanent registrars who would be present at all times in case of any emergencies. Efforts was made to increase the nurse to patient ratio, while a functioning laboratory was developed to fast track test results for critically ill children.

The facility has five patient beds and ~1:1 patient to nurse ratio, a rotating pediatric resident as well as pediatricians conducting ward rounds at least once a day. There is close monitoring of vital signs four-hourly, feeding care, and ambulatory care. The facility is stocked with emergency medications, oxygen concentrators, and conducts a continued training of staff for cardiopulmonary resuscitation amongst other milestones that have been achieved.

Currently, the laboratory services for critically ill children is through the usual route like any other patient, causing delays in patients' management. Radiological tests are done in a remote facility, causing patients to be transferred with a portable oxygen tank, which is distressful for them, their caregivers as well as the health care providers.

The current practice of acute care in Tanzania under the referral system, requires children to first be attended at a primary care office and given pre-hospital care. Thereafter, they are taken to the emergency department where they are resuscitated and then stabilized. Upon stabilization, they are then dispatched to the appropriate units, such as the critical care unit, where a team of trained staff receives them. Here, the child is evaluated and managed based on observation, history, physical examination, and investigations. As soon as the child is stable and out of critical state, he/she is then dispatched to the general wards where continued rehabilitation and preventive education is on-going until the child is fit for discharge.

### Study Population

Participants included all consented caregivers of children (>28 days and <14 years of age) admitted, and all health care providers working in the APCU. Re-admissions within 7 days of discharge and health workers who rotated for <2 weeks in APCU were excluded. As per the guiding standard operating procedures, the APCU grants permission to only one caregiver per child who is admitted for infection control purposes. On average, 30 patients are admitted each month. The estimated sample size for patients admitted over 3 months was therefore 90 participants. Considering all 17 health workers, the minimum sample size was therefore estimated to be 107 participants. Owing to the nature of the study and participants, we used a convenient sampling technique to recruit all participants meeting the selection criteria until we reached the minimum sample size.

## Measurements

The independent variables of interest was quality of care, measured using Quality Improvement of Health Services (QIHS) (10), Observation checklist, Semi-structured questionnaires for both caregivers and Healthcare providers, and an interview guide for Key informant interview. Through these tools, we also measured the structure and the process quality. The outcome variables measured were the patients' outcomes such as survival at discharge from APCU, death, and level of caregiver satisfaction.

The QIHS tool was an adapted form of a checklist from Quality Improvement of Health Services (QIHS) in Tanzania, which was a validated set of indicators for measuring quality of maternal, new born, and child health services as well as related hospital services introduced in July 2015 (10). The set of 306 indicators are categorized into five domains; clinical care, communication, management, people, quality, and safety. The indicators were categorized according to the Donabedian Model including structure (68), process (136), and outcome (102). These indicators were modified according to our settings in all three domains to meet the objectives. Twelve Tanzanian hospitals in Lindi, Mtwara, Mbeya, and Tanga regions had been assessed twice using these indicators (10).

A checklist was adapted from the Quality Improvement of Health Services (QIHS) set of indicators (10), as well as additional requirements according to international pediatrics ICU standards and that of MOHCDGEC (11). Parts of the checklist contributed to developing the semi-structured interviews for feedback about structure.

The caregivers were given a semi-structured questionnaire that was designed based on the adapted form of QIHS set of indicators, observation, and researcher experience. The nursing officers, nursing attendants, and residents were given a different semi structured questionnaire. These questionnaires were designed based on the adapted form of QIHS set of indicators, Consumer Assessment of Health Care Providers and Systems (CAHPS) (12), observation and researcher experience.

A modified interview guide that was being used at MNH for in-patient services was applied for conducting a KII. This was used to gather information on quality of care provided. It also entailed open ended questions that was retrieved from a Consumer Assessment of Health Care Providers and Systems (CAHPS) hospital survey questionnaire titles (12), QIHS list of indicators, experience of researcher, and information from components of the checklist. This created a wholesome interview guide covering all aspects of QOC. Where participants would deviate from the topic, probing open-ended questions were directed toward them. The tools were pre-tested among caregivers attending pediatric outpatient services prior to application. The interviews ended when researchers were satisfied that the saturation point was reached, that is to say, when no new ideas relevant to our study were foreseen (13).

## Data Collection Procedures

Three areas based on the Donabedian model (14) were assessed. In terms of structure, the following was assessed; the presence of favorable infrastructure and environment for health care in APCU, the consistent presence of life-saving drugs and equipment, availability, and presence of qualified human resources i.e., healthcare providers and presence of an up-to-date treatment guideline. This was assessed through observation method. In terms of process, the following was assessed; time to medical attention and intervention, management of emergency situations, inpatient care which would include; appropriate diagnosis and timely management and documentation of patient management as well as staff knowledge and practices which entailed; feedback, education, prevention as well as interaction between healthcare providers and caregiver/patient. In-patient care was assessed by observation of the admission process as well as feedback from the caregivers and healthcare providers (from semi-structured interviews and KIIs). Staff knowledge and practice were assessed by observation of daily patient care and during emergency-situation (such as during CPR). It was also assessed via the semi-structured interviews where open-ended questions were asked about patient care and emergency situations. The outcomes of this study measured the following; the proportion of children who survived to discharge from APCU, proportion of children who died at any point during this study, the median duration of stay in APCU and level of caregiver satisfaction of the QOC provided. This was assessed through document analysis using available records toward the end of the study. In this study we defined septicaemia as a life-threatening organ dysfunction caused by a dysregulated host response to infection (15). Septic shock was also defined as sepsis-induced with hypotension despite adequate fluid resuscitation along with the presence of perfusion abnormalities (15).

During the data collection, two research assistants were recruited to assist the researcher during interviews and observation, for triangulation, to minimize bias. These assistants alternated days and observed at random times of the day to avoid the Hawthorne effect (16) and observer bias. The level of Caregivers satisfaction was evaluated via a semi-structured interview that involved a Likert scale. The interview was conducted outside APCU and after the death or discharge of the patient to allow fading of social desirability. It also encouraged an open and honest communication between researcher and Caregivers.

## Data Management and Analysis

This study employed both quantitative and qualitative methods. For quantitative data, semi structured questionnaires, and document analysis were used for data collection. Data were analyzed using Statistical Package for Social Sciences (SPSS) Version 20 program. Analysis was conducted using descriptive statistics. Continuous variables were expressed as median and interquartile range while categorical data were expressed as proportions. For qualitative data, observations checklist, KIIs, and document analysis were used. KII and contents of the checklist were analyzed using qualitative content analysis with open code software 4.02. Qualitative data analysis was undertaken using descriptive data techniques that allowed depiction of the existing situation within APCU. Themes were developed according to the components of the Donabedian model. This was useful in evaluating the overall QOC of the services provided to address the gaps and the possible ways forward.

Quality assurance is a systematic approach to review the data collected and methods used (17). It identifies possible improvements and ways to bring them about. This was achieved in the following manner:

After the KII, the trained research assistants listened to the recordings of the interview, discussed, and reviewed questions that were difficult to answer and modify them further. Transcription of the KII from the recording device to a word document to ensure consistency was done and re checked for ensuring accuracy and consistency. Data entry and cleaning for quantitative data.

This helped to look for consistency of all entries.

## Ethics Approval and Consent to Participate

Ethical clearance and permission to conduct this study were sought from MUHAS Senate Research and Publications Committee and MNH administration, respectively. Prior to enrolment, the Caregivers and health care providers were informed about the study and consent was attained (verbal and written/signed) and confidentiality was warranted. We invited the identified caregivers and health care providers into the study, explained them in detail about the objectives and their roles in the study. We also informed them of the risks involved and ensured them of the confidentiality of information given. Caregivers were also assured that, refusal to participate would never affect their care, then, and in the future. We also informed them that they can withdraw from the interview or refuse to answer any or part of the questions asked. None of the participants refused consent. This consent included a specific clause allowing the researcher to record parts of the conversations. In sensitive situations such as the death of a child, special ethical measures were sought. All human rights were protected and reserved.

## Ethical Consideration in Sensitive Settings

In the unfortunate scenario where the caregiver had lost their child to death, the research assistant politely requested to speak to the caregiver concerning feedback about their experience. In the case they refused to participate, the research assistant withdrew and did not probe further.

In the case that they agreed to participate, the research assistant proceeded with the semi structured questionnaire. If a point reached and the caregiver was not comfortable to proceed, the research assistant discontinued to probe further. This helped to identify the positives and shortcomings on the care provided i.e., good and bad experiences.

In the case when a life-threatening situation occurred at any point of this study, the researcher or the assistants shouted for help to draw attention to the seniors in the APCU to intervene as soon as possible. If no response, then the researcher or the assistants intervened to save the life of the patient.

## Results

These were summarized into five categories of outcome, structure, process, feedback, and recommendations as guided by the Donabedian model. The outcomes analyzed during this study-encompassed survival to discharge from APCU, death and level of Caregiver satisfaction.

### Descriptive Characteristics

Ninety children were admitted in 3 months duration. Majority of the children were between the ages of 1 month−4 years, 68 (75.6%) with a median age of 2 years.

There were more males 46 (51.1%) compared to females and majority were from within Dar-es-Salaam 70 (77.8%) (Table 1).

**Table 1:** Sociodemographic characteristics admitted children.

	**Male**	**Female**	**Total**
Age (Years) 1 Month-4	33 (71.7%)	35 (79.6%)	68 (75.6%)
5–9	5 (10.9%)	6 (13.6%)	11 (12.2%)
10–14	8 (17.4%)	3 (6.8%)	11 (12.2%)
Domicile		
Dar-es-Salaam	33 (47.1%)	37 (52.9%)	70 (77.8%)
Upcountry	13 (65%)	7 (35%)	20 (22.2%)

Of the total admissions, 66 (73.3%) survived to discharge, while 24 (26.7%) died within APCU. Of those who died, ten (41.7%) passed away in the first 24 h of admission, 14 (58.3%) during the night shift and 20 (83.3%) were a “Transfer in” from other wards. The median duration of stay was 5 days, with a minimum of 4 h and a maximum of 32 days (Table 2).

**Table 2:** Outcomes of children admitted.

	**Male**	**Female**	**Total**
Death	13 (54.2%)	11 (45.8%)	24
Survival to discharge from APCU	33 (50%)	33 (50%)	66
Duration of stay			
<24 h	8 (57.1%)	6 (42.9%)	14
24–48 h	10 (66.7%)	5 (33.3%)	15
48–72 h	6 (60%)	4 (40%)	10
>72 h	22 (43.1%)	29 (56.9%)	51
Readmissions	2 (40%)	3 (60%)	5

**Figure 1:**
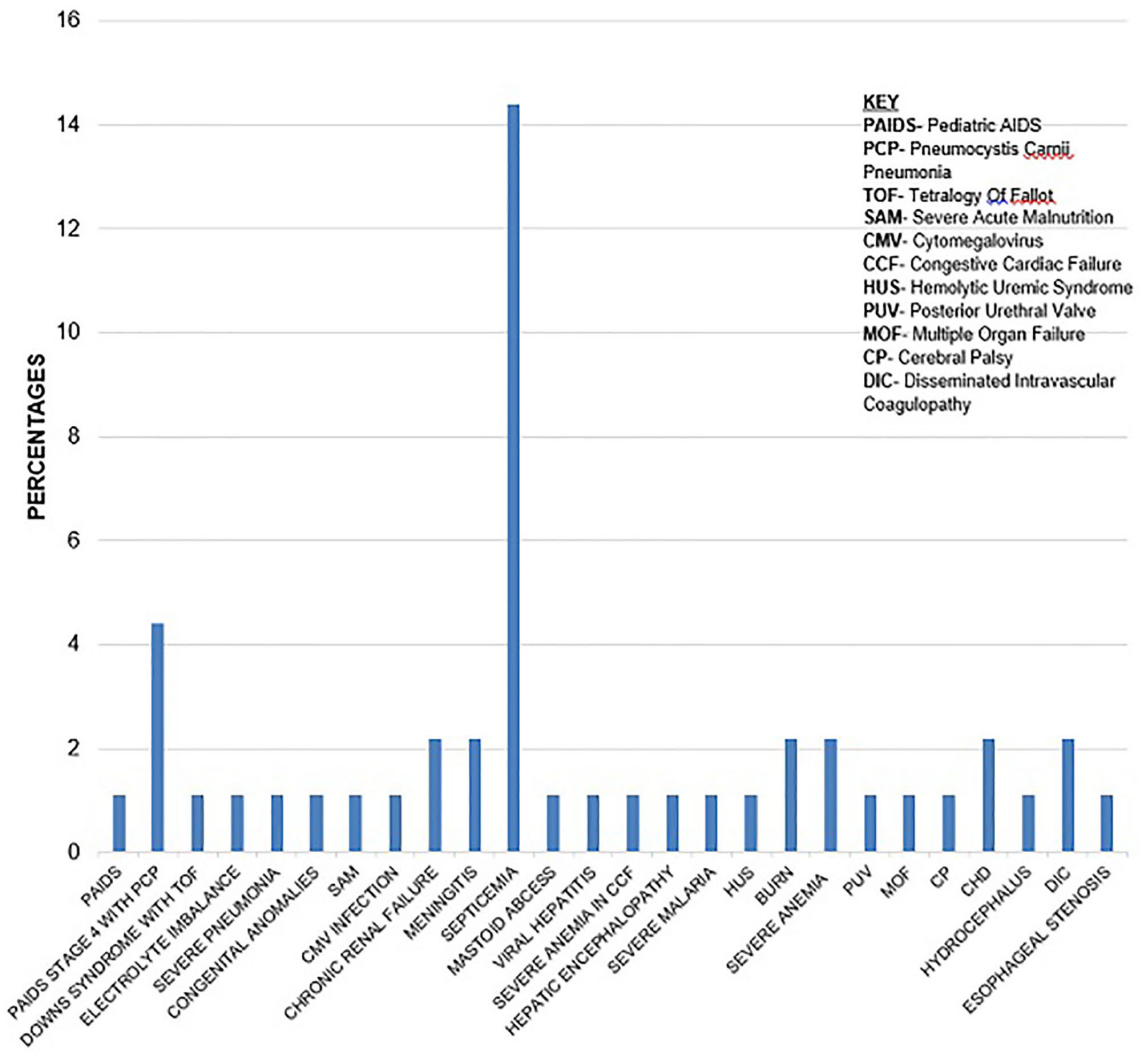
Underlying causes of death.

**Figure 2:**
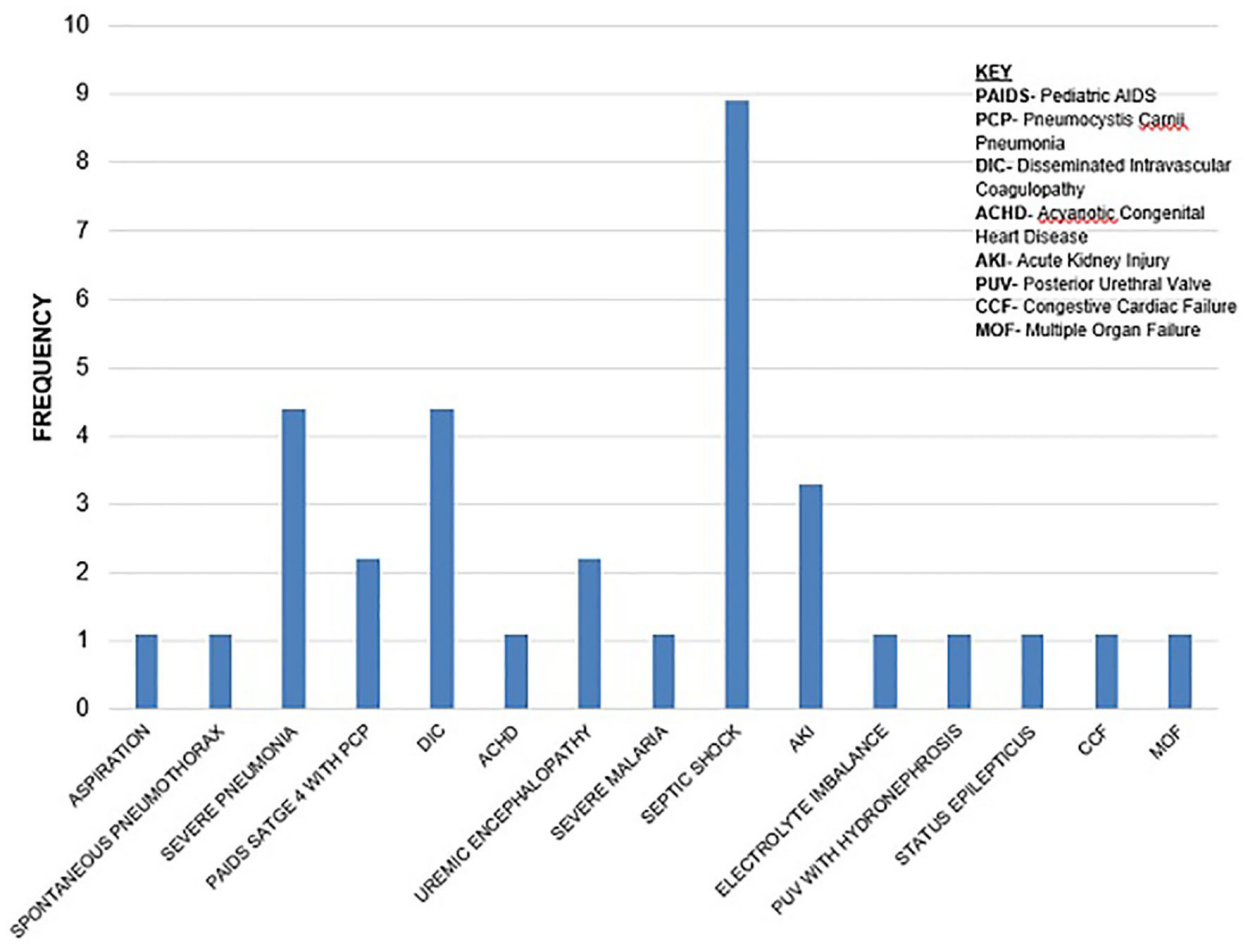
Immediate causes of death.

Most of the underlying causes of death, 17 (70.8%), were infectious with septicaemia 13 (76.5%) being the most common. Septic shock accounted for many 9 (60%) of the immediate causes of death (Figures 1, 2).

### Caregivers Satisfaction

From the caregivers that consented, most reported to be VERY SATISFIED 34 (37.8%) and SATISFIED 22 (24.4%) with the quality of services provided.

**Figure 3:**
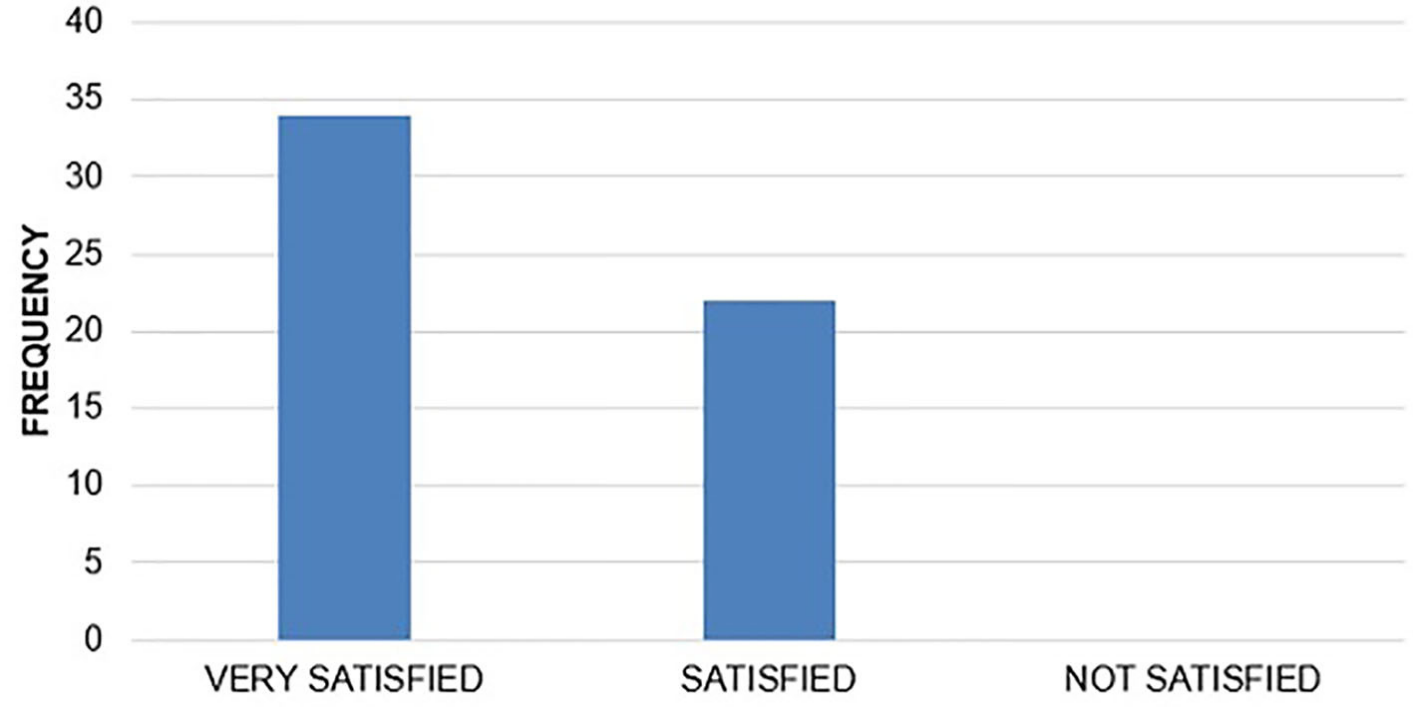
Level of CG satisfaction.

Although, a comparable number 34 (37.8%) of caregivers refused to comment on this (Figure 3).

### Structure

Based on observations made, the physical setting in APCU had the basic requirements for management of critically ill children but lacked in major areas in terms of infrastructure, qualified healthcare providers trained in critical care, updated treatment guidelines, shortage of functional emergency equipment, and emergency drugs. Caregivers reported an unsupportive environment during their stay.

“*…I urge the APCU to create a self-contained toilet and bathroom services, as we need to walk a long distance and a great deal of time is spent away from our sick children as we need to go outside the APCU to be able to get access to toilets.”* (Caregiver)

When compared to the international PICU and MOHCDGEC requirements, the current setting in APCU was lacking in several pertinent areas.

Availability of adequate and qualified human resources in critical care was a challenge faced in our setting in APCU.

“*…they need to increase the number of health working staff, human resource is not adequate”* (Caregiver)

The treatment guideline used in APCU were last update in 2005.

### Process Indicators

**Figure 4:**
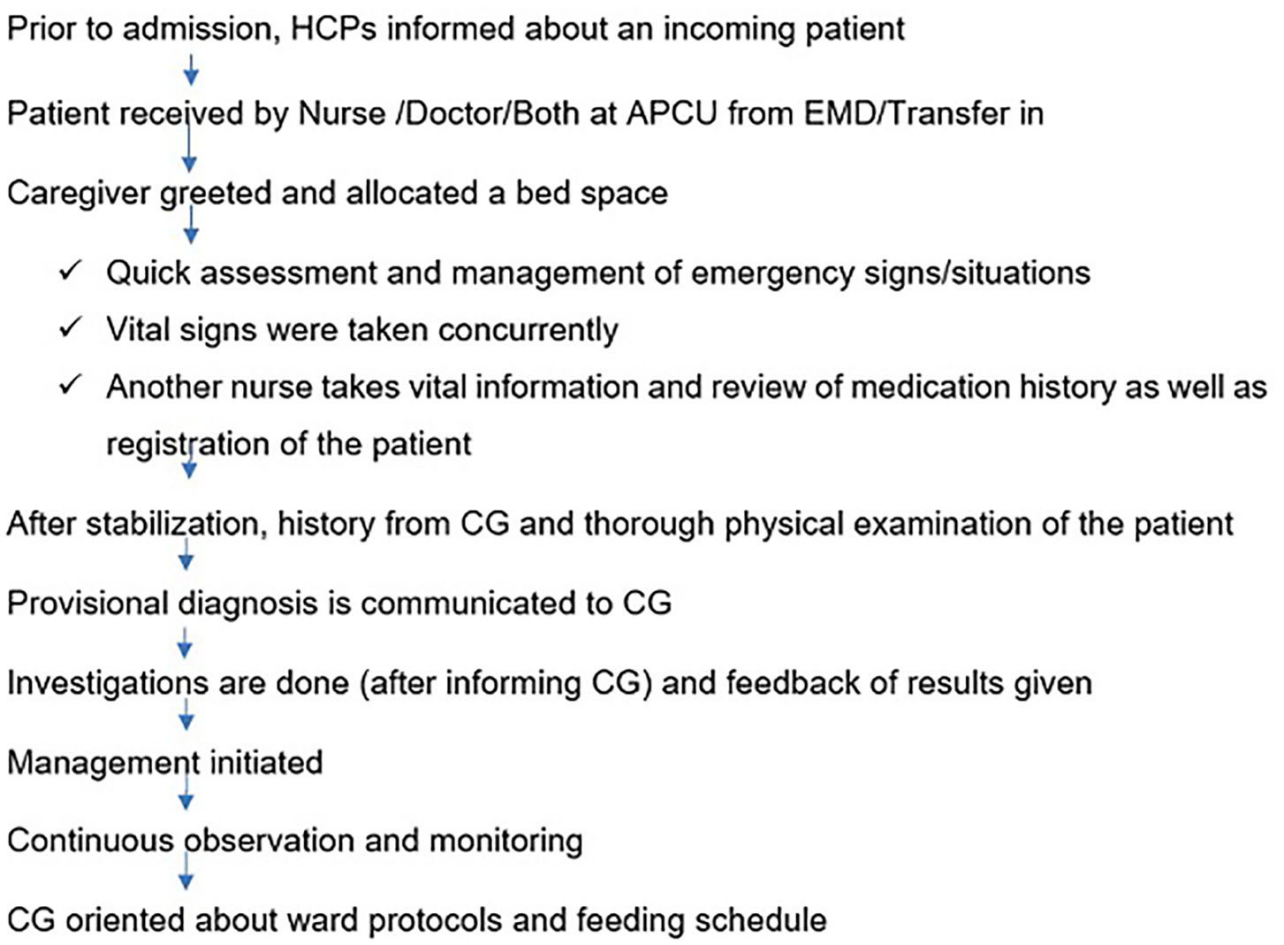
Admission process.

The admission process was noted to be thorough and consistent for every patient that arrived at APCU. Some setbacks were noted. Delays in the referral process and health worker training were amongst some matters that required attention (Figure 4).

“*…I am so proud of services at MNH, I didn't imagine that during the night the nurses and doctors will be available. I think the services are good and satisfactory.”*(Caregiver)

Delay in the referral system proved a frustration to both the Caregivers and healthcare providers as confirmed during interviews and KIIs, respectively.

“*….In terms of process, the standards of health care services are not met. The biggest challenge we face is the delay in referral system or transferring process of patients from other wards. Due to this delay, the interventions put in place to save a child's life tend to fail, as it is too late. Thus there needs to be a better referral/transferring system in place so as to enable early interventions and a possible change in outcome…"*[Key Informant (KI)]

The documentation process was thorough and consistent. Procedures during resuscitation were reported with findings. Death certification was also well-documented though the immediate and underlying causes of death were not indicated immediately post resuscitation. It was noted that none of the healthcare providers in APCU have been formally trained in pediatric critical care.

### Feedback Process

This had major discrepancies between Caregivers and healthcare providers especially during emergencies and death of a patient despite there being a good communication between the two groups.

“*…I was not informed about my child's prognosis… I knew my child had a kidney problem and was given hope that a person can survive with one kidney, so I had hope that my child will be operated and would recover.”* (Caregiver).

## Discussion

One out of four children admitted in APCU at MNH died. A significant proportion of them died within the first 24 h of admission. These high mortality rates are comparable to other contexts. The mortality rates in our setting are much lower from the projected picture in African countries (2). Although comparison with such studies is challenging. Speculated reasons could be that the APCU is a unit that provides centralized medical care. It is not a shared or mixed facility with other sub specialities such as surgical or neonatal ICU. The age limitation in APCU restricts the admission of neonates that require specialized “high care,” observation and treatment thus reducing the mortality rate all together. In addition, the APCU admits an average of 30 patients per month. This is number is much lower compared to the admissions in other PICUs in other developing countries. Disease patterns, geographical location, health-seeking behavior, access to medical care and differences in management protocols vary, amongst other possible reasons.

The fact that more than thirty percent of the caregivers did not comment on the level of satisfaction can be extrapolated in many different ways. It can be attributed to the fact that despite reassurance, as standard of care during requesting consent, these caregivers had inherent worries on how their responses would affect the quality of care their children would receive. Social desirability is another factor that could explain this response.

A glance on the impact of structure on QOC in APCU; the strengths of the structural analysis revealed that the APCU infrastructure existed with a management plan. However, additional improvements need to be made in terms of infrastructure, workforce, updated treatment guidelines, trained personnel in critical care and emergency functional equipment amongst others. Training in pediatric basic life support (PBLS) was observed amongst four (23.5%) out of the seventeen health care providers (last upgraded in 2016). Since health care providers do all of the resuscitations, it is mandatory that they are updated regularly on the current triage and resuscitation techniques so as to improve the quality of the resuscitations and eventually the outcome of these patients (18).

The working environment and infrastructure need to improve to motivate the health care providers to provide a better quality of services. As one of the observed factors that brought discouragement to the health care providers (19), it was replicated in the feedback from the caregivers as well. The environment in APCU was unfavorable to the participants, which led to discomfort during their stay. In terms of infrastructure, several areas were noted to be lacking when compared to the international PICU requirements and standards set by the MOHCDGEC (11). This included lack of nearby pediatrician call rooms, lack of laboratories for emergency tests, difficult adjustable head beds for resuscitation, lack of dedicated pediatric intensivists, and shortage of trained and qualified staff amongst others. Although we belong in a resource-limited setting, certain standard requirements are set to improve the quality of services provided and thus impacting the outcomes.

Burnout rates were noted to be high amongst the health care due to shortage of human resources at all levels of care in APCU. This has led to an increased level of stress, lack of motivation as well as lack of job satisfaction (20).

From observation, the current treatment guideline used in APCU was last updated in 2005, which is 13 years ago. The current guidelines, although stand the test of time, is constantly challenged by the updated management and advancement in medicine. Updated versions are required for the inexperienced as well as for adding knowledge to the existing team of health care providers.

The admission process had several commendable points and setbacks. Patients were evaluated immediately upon arrival and assessed by the entire team. Time to response during emergencies was immediate, although it was noted there was a lack of feedback regarding the patient condition during this time. The staff were conversant on matters of daily in-patient care and emergencies although they required refresher-training courses in PBLS and Pediatric Advanced Life Support (PALS). Although the response time during emergencies was immediate, the outcomes to CPR done was death. This has led to questioning the quality of CPR delivered to these patients (21). The lack of availability of functional equipment as well as emergency drugs, contributed to poor quality of CPR.

Delays in the referral system was noted by caregivers, and was a major setback in the QOC (22). The delay may have been from the peripheral hospitals or health seeking behavior of caregivers. This can be related to the domicile as a proxy of the level of education of those caregivers. Majority of the patients were from within Dar-es-Salaam, mostly Mbagala and Kigamboni, where a majority are suspected to have a primary level of education. Training of doctors in peripheral hospitals for management of common childhood illnesses needs to be ongoing and updated. This would reduce the burden on tertiary hospitals in general.

The documentation process was thorough and systematic although the immediate and underlying causes of death were not documented instantaneously, affecting the feedback process.

Despite a good communication between the health care providers and the Caregivers, there was a vast discrepancy in views regarding feedback process. The health workers need to be trained further on imparting bad news, and ways of dealing with families who have lost their child to death (23, 24).

### Study Limitations

Results obtained in this study needs to be carefully discussed owing to the following potential limitations. The administrative personnel were not included in this study and could have served as a proxy of the challenges encountered by the Health care providers in APCU. Upon admission into APCU, the frequency of specialist review within the initial 24 h was overlooked and thus would have been a proxy of the care that the child received. This study was conducted at a referral teaching hospital, thus the results obtained cannot be generalized to other relatively smaller facilities. They do not reflect the QOC in Tanzania as a whole, but in facilities with similar context as MNH. The outcome of survival to discharge from APCU was at a point in time and therefore it was not possible to follow up on adverse/long-term outcomes of these patients.

## Conclusions

One in four children admitted in APCU died, many of them within the first 24 h. The risk of death was high during the night shift and among the children transferred from other wards. These deaths reflect a weakness in the decision-making and delays thereof; shortage of staff, especially during the night shift; quality of services provided; lack of updated guidelines; and lack of refresher courses in PBLS and PALS.

## Recommendations

In addressing mortality in APCU at MNH and units with similar context, there is a need to update the treatment guideline for APCU. Measures of achieving an equal ratio of staff to patient, especially during the night shift, is highly advisable. In order to obtain better, functional and user-friendly modern equipment for the APCU, they should be maintained regularly. Each bed must have a set of basic emergency equipment.

Since APCU is part of MNH, a tertiary referral hospital, it is recommended that its infrastructure needs to be upgraded to meet the international standard requirements of a PICU. Continuous Medical Education of all the staff concerned needs to be mandatory, especially training in PBLS and PALS.

## Data Availability Statement

The datasets generated for this study are available on request to the corresponding author.

## Author Contributions

AR contributed in conceptualization, selection of study design, acquisition, analysis and interpretation of data as well as development, and review of final manuscript for submission. AM participated in conceptualization and review of manuscript for final submission. KM contributed in conceptualization, analysis and interpretation as well as review of the final manuscript for submission. NS has participated in conceptualization, designing, analysis and interpretation of data as well as review of the final manuscript for submission. AA participated in conceptualization, analysis, and review of the final manuscript for submission. BS participated in analysis and review of final manuscript for submission. All authors read and approved the final manuscript.

### Conflict of Interest

The authors declare that the research was conducted in the absence of any commercial or financial relationships that could be construed as a potential conflict of interest.

## Abbreviations

APCU: Acute Paediatric Care Unit
CAHPS: Consumer Assessment of Health Care Providers and Systems
CG: Caregiver
DSM: Dar-es-salaam
ETAT: Emergency Triage Assessment and Treatment
HCP: Health Care Providers
ICU: Intensive Care Unit
KII: Key Informant Interview
MUHAS: Muhimbili University of Health and Allied Sciences
MOH: Ministry of Health
MNH: Muhimbili National Hospital
MOHSW: Ministry of Health and Social Welfare
MOHCDGEC Ministry of Health, Community Development, Gender: Elderly and Children
PICU, Paediatric Intensive Care Unit, PBLS: Paediatric Basic Life Support
PALS: Paediatric Advanced Life Support
QIHS: Quality Improvement of Health Services in Tanzania
QOC: Quality of Care
TZ: Tanzania
WHO: World Health Organization.
